# Comparative Genomics of the Herbivore Gut Symbiont *Lactobacillus reuteri* Reveals Genetic Diversity and Lifestyle Adaptation

**DOI:** 10.3389/fmicb.2018.01151

**Published:** 2018-06-04

**Authors:** Jie Yu, Jie Zhao, Yuqin Song, Jiachao Zhang, Zhongjie Yu, Heping Zhang, Zhihong Sun

**Affiliations:** ^1^Key Laboratory of Dairy Biotechnology and Engineering, Ministry of Education, Inner Mongolia Agricultural University, Hohhot, China; ^2^Key Laboratory of Dairy Products Processing, Ministry of Agriculture, Inner Mongolia Agricultural University, Hohhot, China

**Keywords:** *Lactobacillus reuteri*, herbivore, comparative genomics, host-specific gene, cell adhesion, carbohydrate active enzymes

## Abstract

*Lactobacillus reuteri* is a catalase-negative, Gram-positive, non-motile, obligately heterofermentative bacterial species that has been used as a model to describe the ecology and evolution of vertebrate gut symbionts. However, the genetic features and evolutionary strategies of *L. reuteri* from the gastrointestinal tract of herbivores remain unknown. Therefore, 16 *L. reuteri* strains isolated from goat, sheep, cow, and horse in Inner Mongolia, China were sequenced in this study. A comparative genomic approach was used to assess genetic diversity and gain insight into the distinguishing features related to the different hosts based on 21 published genomic sequences. Genome size, G + C content, and average nucleotide identity values of the *L. reuteri* strains from different hosts indicated that the strains have broad genetic diversity. The pan-genome of 37 *L. reuteri* strains contained 8,680 gene families, and the core genome contained 726 gene families. A total of 92,270 nucleotide mutation sites were discovered among 37 *L. reuteri* strains, and all core genes displayed a *K*_a_/*K*_s_ ratio much lower than 1, suggesting strong purifying selective pressure (negative selection). A highly robust maximum likelihood tree based on the core genes shown in the herbivore isolates were divided into three clades; clades A and B contained most of the herbivore isolates and were more closely related to human isolates and vastly distinct from clade C. Some functional genes may be attributable to host-specific of the herbivore, omnivore, and sourdough groups. Moreover, the numbers of genes encoding cell surface proteins and active carbohydrate enzymes were host-specific. This study provides new insight into the adaptation of *L. reuteri* to the intestinal habitat of herbivores, suggesting that the genomic diversity of *L. reuteri* from different ecological origins is closely associated with their living environment.

## Introduction

*Lactobacillus reuteri* is a catalase-negative, Gram-positive, non-motile, obligately heterofermentative bacterial species that is frequently found in the gastrointestinal (GI) tract of humans and other animals, sourdough, and meat products. Numerous studies have documented that *L. reuteri* exhibits various probiotic effects in humans and animals, including the ability to colonize the intestine, modulate the host’s immune system (Valeur et al., 2004), produce and excrete broad spectrum antimicrobial compounds (Talarico and Dobrogosz, 1989), prevent diarrhea (Weizman et al., 2005; Kołodziej and Szajewska, 2017), reduce infant colic (Indrio et al., 2008), and prevent colitis (Schreiber et al., 2009). In addition, *L. reuteri* has been used as a model to describe the ecology and evolution of vertebrate gut symbionts (Walter and Klaenhammer, 2011; Duar et al., 2017). Therefore, the growth, metabolism, and evolution of *L. reuteri* have been extensively studied.

Oh et al. characterized the phylogeny and population genetic structure of *L. reuteri* strains isolated from omnivores (human, mouse, rat, pig, chicken, and turkey) using the amplified-fragment length polymorphism and multi-locus sequence analysis techniques. The results showed that considerable genetic heterogeneity exists within the *L. reuteri* population, with distinct phylogenetic clades reflecting the host origin of the strains (Oh et al., 2010). Subsequently, the phylogenetic analysis and evolutionary trajectories of porcine *L. reuteri* strains were analyzed by comparative genomics (Wegmann et al., 2015). The results reflect the genomic events in *L. reuteri* that occur during specialization to their hosts, and genes of encoded cell surface proteins may contribute to the occurrence of two distinct pig-derived clades and their observed host specificity. The evolution and ecology of *L. reuteri* in the animal GI tract has been analyzed using a genomic approach and animal model experiments to further validate adaptation of host-specific strains. Reports have demonstrated host adaptation of *L*. *reuteri* strains, as only rodent strains colonize mice (Frese et al., 2011) and poultry strains colonize chickens efficiently, whereas experiments in humans and pigs did not provide evidence of adaptation of *L*. *reuteri* to these hosts (Duar et al., 2017). Despite these advances, the evolutionary strategies of *L*. *reuteri* strains isolated from the GI tracts of herbivores remain unknown, and the ecological relationships between herbivorous intestinal isolates and omnivorous intestinal isolates are unclear.

Comparative genomic studies of strains within the same species have provided insight into modified, acquired, or lost genetic features that have facilitated the evolution and adaptation of strains to specific environmental niches (Francis et al., 2013). In recent years, along with the rapid development of throughput DNA sequencing technologies, genomic studies have become more feasible and affordable, and genomic data of numerous organisms have become publicly available. Until now, more than 90 *L*. *reuteri* strains isolated from humans, mice, rats, pigs, chickens, and sourdough have been fully sequenced and published in the National Center for Biotechnology Information Center as of April 2018. However, few *L*. *reuteri* strains from the GI tract of herbivores have undergone whole-genome sequencing. Therefore, 16 herbivore-derived *L*. *reuteri* strains isolated from Inner Mongolia, China were sequenced in this study. We used a comparative genomic approach to define the pan-genome, core genome, and unique genes of the isolates from herbivores; assessed genetic diversity and gained insight into the distinguishing features; and compared the numbers of genes encoding adhesion proteins and carbohydrate-active enzymes (CAZymes).

## Materials and Methods

### Genome Sequencing, Annotation, and Average Nucleotide Identity Calculation

The 16 *L*. *reuteri* isolates used in this study were isolated from feces of different hosts, including goats, sheep, cows, and horses in Inner Mongolia, China. Strain information is listed in **Supplementary Table S1**. These isolates were cultured in MRS broth (Oxoid Ltd., Basingstoke, United Kingdom) at 37°C for 24 h. Genomic DNA was extracted using the TIANamp Bacteria DNA Kit (Tiangen, Beijing, China) in accordance with the manufacturer’s instructions. Concentration and purity were verified with a NanoDrop spectrophotometer (NanoDrop Technologies, Inc., Wilmington, DE, United States). Whole-genome sequencing was performed using the Illumina HiSeq platform (Illumina, Inc., San Diego, CA, United States), generating 150 bp paired-end reads per sample. Before assembling the sequences, adaptor sequences, low quality sequences, and successive N sequences of the raw data were filtered from the Illumina HiSeq system. The high quality sequences were assembled using SOAPdenovo2 (Luo et al., 2012) on a 64-bit Linux system. The CheckM software was used to check the completeness and contamination in the genome sequences (Parks et al., 2014). The genome sequences of 21 strains isolated from feces of humans, pigs, mice, rats, breast milk of humans, and sourdough published previously were acquired from GenBank to compare the different host-derived strains. All genome sequences of the *L*. *reuteri* strains were annotated by uploading each sequence file to the Rapid Annotation using Subsystem Technology (RAST) web server (Aziz et al., 2008) for genomic annotation. The protein coding sequences were predicted and categorized based on the SEED subsystem (most prominently FIGfams) in RAST. Additionally, COG annotation was carried out with the COG (Tatusov et al., 2003) database by blastx (Altschul et al., 1990) using an e-value cut-off of 1e-6.

To evaluate the genetic relatedness between the reference genome *L*. *reuteri* DSM20016 and the 37 strains of *L*. *reuteri*, the average nucleotide identity (ANI) was calculated based on the JSpecies package^1^ (Richter and Rosselló-Móra, 2009) and the method proposed by Goris et al. (2007). The ANI value between the query and reference genome was calculated as the mean identity of all BLASTN matches that showed more than 30% overall sequence identity (recalculated to an identity along the entire sequence) over an alignable region of at least 70% of their length.

### Core Genome, Pan-Genome, Unique Gene, and Phylogenetic Analysis

The nucleotide sequences of the 37 *L*. *reuteri* strains were first annotated using Prokka v1.12 (Seemann, 2014) to obtain GFF formatted files, which were then used to calculate the core- and pan-genomes. The core- and pan-genomes were calculated using Roary (Page et al., 2015), which is a rapid standalone pan genomic pipeline. A pair of genes was defined as belonging to the same gene family when the identity value of their amino acid sequences was >95%. In addition, a maximum likelihood (ML) tree was constructed based on the core genes. Host-specific genes were showed in Venn plot, which was constructed by Venny 2.1 (Oliveros, 2007)^2^. The unique genes were confirmed by BLASTN. The genome of 37 strains were blast to unique genes with default parameters. The identity above 95% was considered as gene presence, while identity below 50% was considered as gene absence. The identity of each unique gene was showed by heatmap. Unique genes were annotated by KEGG automatic annotation server^3^.

### Variation Analysis and Profiling of Adhesion and Active Carbohydrate Enzyme Genes

To study polymorphism and the evolutionary rate of *L. reuteri* genes, we identified single nucleotide polymorphisms (SNPs) in the draft genome sequences of the 37 *L*. *reuteri* genomes, in which *L*. *reuteri* DSM20016 was considered the reference genome. SNPs were identified by aligning scaffolds of each strain to the reference genome using MUMmer 3.0 (Kurtz et al., 2004). The MUMmer results for each strain were filtered to remove SNPs that might be unreliable according to the following criteria: (1) quality score <20 (average base calling error rate >0.01); (2) covered by <10 paired-end reads; (3) in repetitive regions; and (4) not identified by BLAST searches of the contigs of each strain to the core genome sequences. In addition, the *K*_a_/*K*_s_ value of each single copy gene was calculated using the KaKs_Calculator^4^ (Wang et al., 2010) and ParaAT^5^ software.

Protein-encoding genes associated with cell adhesion were identified from the gene annotation and prediction results. The sequences were detected and assigned to families of CAZymes using HMMSCAN (from the HMMER package v3.1b1^6^) (Sun et al., 2015). HMMSCAN was applied to predict potential families of glycosyltransferases (GTs), glycoside hydrolases (GHs), carbohydrate esterases (CEs), polysaccharide lyases (PLs), auxiliary activity (AA), and carbohydrate-binding modules (CBMs) across the 37 genomes below a threshold cutoff of 1e-05.

### Statistical Analyses

Differences in the functional gene counts for each isolate were identified with the Mann–Whitney and Kruskal–Wallis tests. Copy numbers of the verified GH, GT, CE, PL, AA, and CBM families as well as the adhesion genes were produced with GraphPad Prism 6 tool (GraphPad Software Inc., La Jolla, CA, United States) and the R package (R Project for Statistical Computing, Vienna, Austria).

## Results

### General Genomic Characteristics of the *L*. *reuteri* Strains

Across the 16 *L*. *reuteri* strains in this study, total genome size varied from 2.14 to 2.47 Mb, with a mean value of 2.28 Mb. In addition, the G + C content ranged from 38.17 to 38.63%, with an average value of 38.39% (**Supplementary Table S2**). Mapped to the publicly available *L*. *reuteri* DSM20016 reference genome, the pairwise ANI value between the strains was >95%, which is the cut-off value to distinguish different species (Goris et al., 2007). The ANI result revealed that they are the same species (**Supplementary Figure S1**). Genome annotation by the RAST server showed that the 16 sequencing genomes and 21 reference genomes of *L*. *reuteri* strains encoded 1,385 (983–1,540) protein-coding sequences (except pseudogenes). The largest proportion of protein coding categories was “protein metabolism” (12.62%) followed by “amino acid derivatives” (10.83%), “carbohydrates” (10.66%), and “cofactors, vitamins, prosthetic groups, and pigments” (10.13%) (**Supplementary Figure S2**).

### Core Genome, Pan-Genome, and Host-Specific Genes Analysis

**FIGURE 1:**
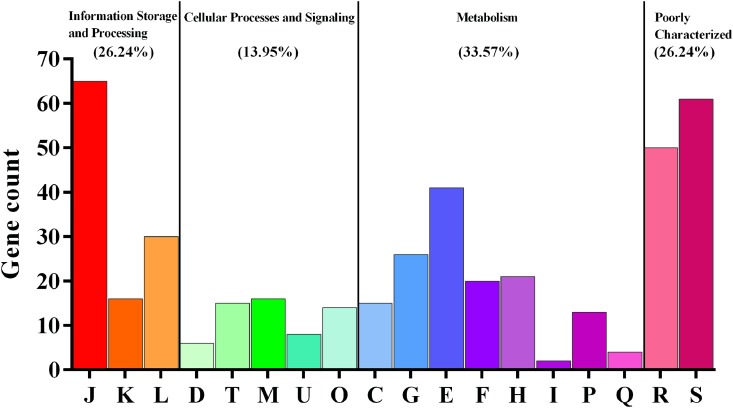
Core genes of *Lactobacillus reuteri* using the COG database. J, translation, ribosomal structure and biogenesis; K, transcription; L, replication, recombination and repair; D, cell cycle control, cell division, chromosome partitioning; T, signal transduction mechanisms; M, cell wall/membrane/envelope biogenesis; U, intracellular trafficking, secretion, and vesicular transport; O, posttranslational modification, protein turnover, chaperones; C, energy production and conversion; G, carbohydrate transport and metabolism; E, amino acid transport and metabolism; F, nucleotide transport and metabolism; H, coenzyme transport and metabolism; I, lipid transport and metabolism; P, inorganic ion transport and metabolism; Q, secondary metabolites biosynthesis, transport and catabolism; R, general function prediction only; S, function unknown.

The core- and pan-genomes of the 37 *L*. *reuteri* strains were defined by a comparative genomics method. The pan-genome of the 37 *L*. *reuteri* strains contained 8,680 gene families, and *L*. *reuteri* had a large open pan-genome whose size increased continuously with the number of added genomes (**Supplementary Figure S3**). In contrast to the pan-genome, the core genome contained 726 gene families and gradually stabilized, indicating that the 37 genomes were sufficient to represent the core genome of *L*. *reuteri.* In addition, the 37 *L*. *reuteri* genomes contained 4,773 accessory gene families. To determine the functions of the 726 core genes, we extracted the sequences of the core gene to map to the COG database. The results showed that the majority encoded essential proteins for metabolism (33.57%) (**Figure 1**). Except for poorly characterized categories, the largest proportion of core genes belonged to the categories “translation, ribosomal structure and biogenesis (J)” (15.37%), followed by “amino acid transport and metabolism (E)” (9.69%).

**FIGURE 2:**
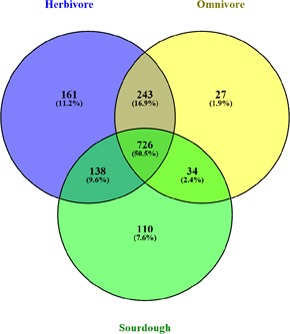
Venn plot showing shared and unique core genes distribution among *Lactobacillus reuteri* strains in herbivore, omnivore, and sourdough groups.

Depending on the source, 37 isolates were divided into three subpopulations, including herbivore group, omnivore group, and sourdough group. We further analyzed the host-specific genes in different subpopulation (**Figure 2** and **Supplementary Tables S3, S4**). The results showed 161 genes were specific in the herbivore group, which were associated with porphyrin and chlorophyll metabolism, biosynthesis of secondary metabolites, biosynthesis of antibiotics, pyruvate metabolism, glycolysis/gluconeogenesis, carbon metabolism, and other biosynthetic or metabolic pathways. Twelve genes that participated in the biosynthesis of secondary metabolites in herbivore group were different from those of the omnivore group and sourdough group, in which six genes and four genes were specific, respectively. Six genes, which participated in amino acid biosynthesis, in herbivore group were differed from the omnivore group, in which only one gene was specific. Six genes associated with pyruvate metabolism, in herbivore group were different from the sourdough group, in which one gene was specific. Specifically, acetate, acetaldehyde, (S)-malate, and acetyl-CoA were produced by acetaldehyde dehydrogenase, acylphosphatase, acetate kinase, and malate dehydrogenase, respectively, whose encoding genes were unique in herbivore group.

### Phylogenetic Analysis and Unique Genes in the Clades of Herbivore-Derived *L. reuteri* Strains

**FIGURE 3:**
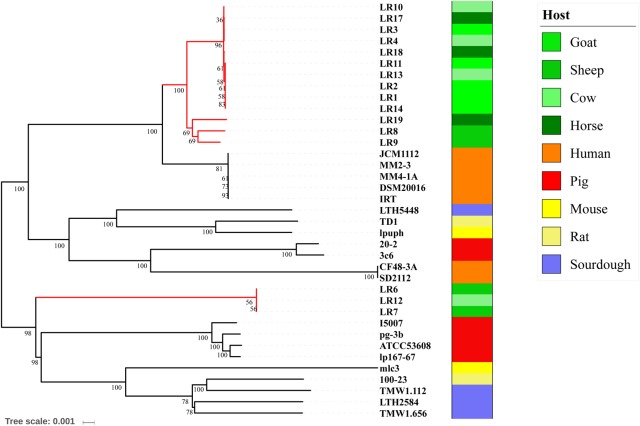
Phylogeny of *Lactobacillus reuteri* was inferred using a maximum likelihood method.

To infer the phylogenetic relationships among the 37 *L*. *reuteri* strains, we constructed a highly robust ML tree based on the core gene sequences (**Figure 3**). The structure of the tree was the same as that of a previous report. All *L*. *reuteri* isolates were divided into two branches, in which clades III, IV, and C were distinct from the other clades. Pig isolates ATCC 53608, lp167-67, pg-3b, and I5007 were in the same clade, which was more closely related to cluster III and cluster C, while pig isolates 20-2 and 3c6 (clade V) were more closely related to clades I, II, VI, A, and B, which contain human and herbivore isolates. Branch A contained five goat isolates, three cow isolates, and two horse isolates. Branch B contained two sheep isolates and one horse isolate. Branch C contained two sheep isolates and one cow isolate and was vastly distinct from branches A and B, all of which were herbivore isolates. These results indicate that different selective pressures may have led to their separation.

**FIGURE 4:**
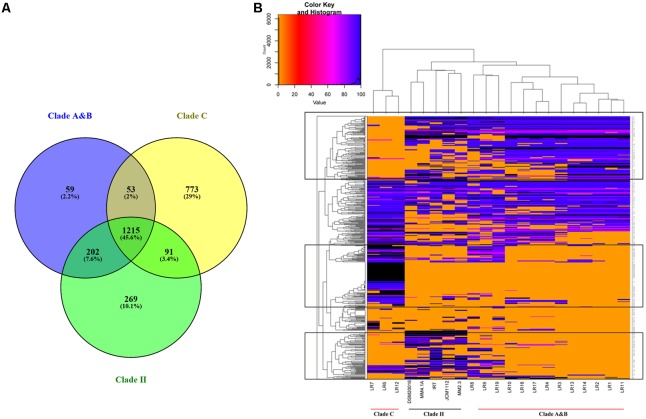
Shared and unique gene families of *Lactobacillus reuteri* strains from different clades of herbivore-derived *L. reuteri* strains **(A)**. Venn plot showing shared and unique genes distribution **(B)**. A heatmap representation of BLASTN comparison of specific genes against sequenced *L. reuteri* genomes.

To further understand the functional genes specific to the individual clades of herbivore-derived *L. reuteri* strains, a Venn plot was constructed based on the core genes in clade A and B, clade C, and clade II (**Figure 4**). The results showed that 864 genes, which were associated with porphyrin and chlorophyll metabolism, atrazine degradation, phenylalanine metabolism, histidine metabolism, and any other metabolic pathways, were included exclusively in clade C, whereas 261 genes, associated with folate biosynthesis, carbon metabolism, quorum sensing, polyketide sugar unit biosynthesis, streptomycin biosynthesis, and other biosynthetic or metabolic steps were included exclusively in the herbivore clade. Thirteen genes that participated in the two-component system in strains of clade C were different from those of the herbivore clade, in which five genes were specific. Eleven genes, which participated in amino acid biosynthesis, in clade C were different from the herbivore clade, in which six genes were specific. Specifically, serine is translated to pyruvate by L-serine dehydratase, whose encoding genes were unique to clade C. Some specific genes associated with isoleucine, arginine, proline, and glutamine biosynthesis were unique to clade C, while some unique genes in the herbivore clade participated in the histidine and methionine biosynthetic pathway. D-Lactate dehydrogenase, water dikinase, and malate dehydrogenase encoding genes were unique to clade C and were associated with the production of D-lactate, phosphoenolpyruvate, and (S)-malate, respectively. In addition, both clade C and the herbivore clade had their own unique genes related to metabolic pathways, biosynthesis of secondary metabolites, antibiotics, and peptidoglycans, microbial metabolism in diverse environments, and purine metabolism (**Supplementary Tables S5, S6**).

The closest lineages to the herbivore clade were clade II, in which the isolates were all from the human intestine. Thus, we further identified the host-specific genes between the herbivore and human clade. There were 1,417 conserved genes in isolates of both the human and herbivore clade; 360 unique genes were in the human clade, while 112 unique genes were in the herbivore clade. The unique genes in the human clade were related to starch and sucrose metabolism, lysine biosynthesis, beta-lactam resistance, amino sugar and nucleotide sugar metabolism, streptomycin biosynthesis, and galactose and other amino acid metabolism, whereas the unique genes in the herbivore clade were associated with glycolysis/gluconeogenesis, carbon fixation in photosynthetic organisms, atrazine degradation, propanoate metabolism, cationic antimicrobial peptide (CAMP) resistance, methane metabolism, and inositol phosphate metabolism. D-Lactate dehydrogenase-encoding genes were unique to the human clade and were associated with the production of D-lactate, whereas L-lactate dehydrogenase and malate dehydrogenase encoding genes only existed in the herbivore clade, which can transform pyruvate to L-lactate and (S)-malate, respectively (**Supplementary Tables S7, S8**).

### Variation Analysis

**FIGURE 5:**
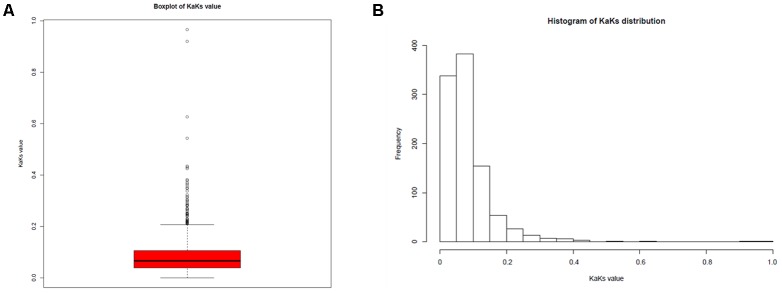
*K*_a_*/K*_s_ values for each gene. **(A)** Boxplot of *K*_a_*/K*_s_ values; **(B)** distribution of *K*_a_*/K*_s_ values.

To illuminate the evolution of the 37 *L*. *reuteri* isolates originating from different hosts, including humans, pigs, mice, rats, cows, horses, goats, sheep, and sourdough, we performed SNP calling to determine the polymorphic sites in the genomes of the *L*. *reuteri* strains. A total of 92,270 nucleotide mutation sites were discovered among the 37 *L*. *reuteri* strains; 18,263 were non-synonymous mutations (19.79%) and 67,151 were synonymous mutations (72.88%) (**Supplementary Table S9**). Almost 100 nucleotide mutation sites per 1 kb were detected in the genome, and some gene regions had up to 200 nucleotide mutation sites, excluding the repetitive domain (**Supplementary Figure S4**). We further calculated the *K*_a_*/K*_s_ value of each core gene in 37 strains (**Figure 5**) and 16 herbivore strains, respectively. The results show that most *K*_a_*/K*_s_ values were <1, indicating that the all *L*. *reuteri* strains and herbivore strains are under negative selection or purifying selection.

### Cell Adhesion

**FIGURE 6:**
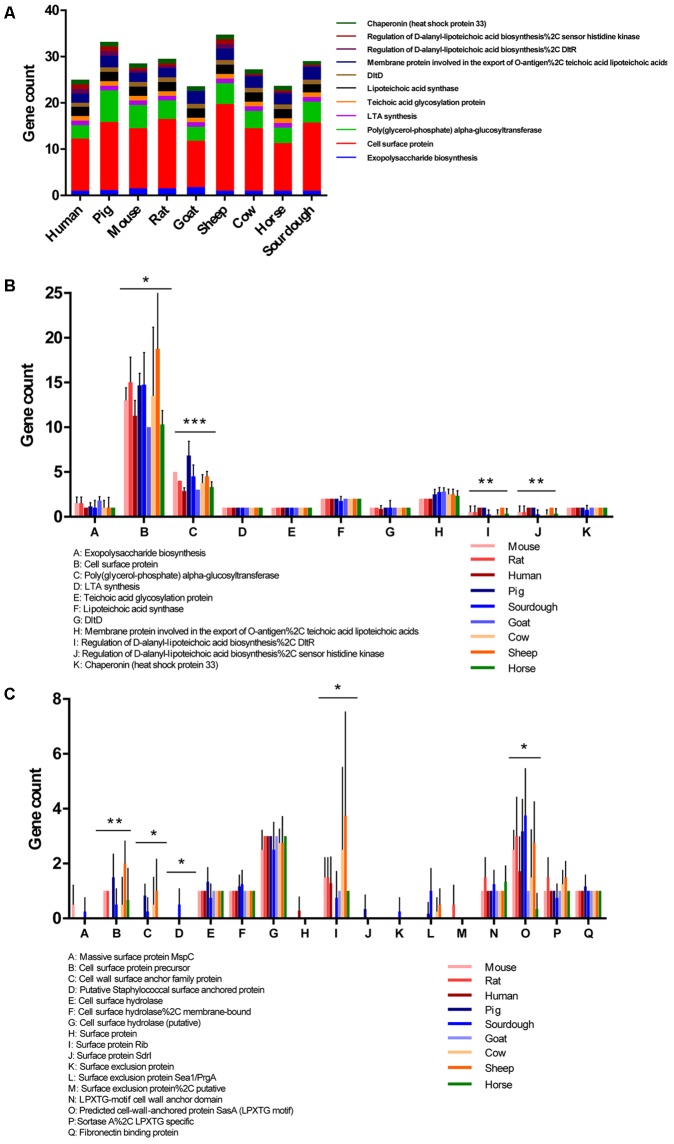
Distribution and abundance of genes related to epithelial adhesion in nine host isolates. **(A)** Total count of adhesion genes in the nine host isolates. **(B)** Differential abundance of adhesion genes in strains isolated from nine isolates. **(C)** Differential abundance of cell surface protein-encoding genes in strains isolated from nine hosts. ^∗^*p* < 0.05, ^∗∗^*p* < 0.01, ^∗∗∗^*p* < 0.001.

An average of 29 genes related to epithelial adhesion were identified in the 37 *L*. *reuteri* genomes isolated from nine hosts. We found 11 gene families involved in exopolysaccharide biosynthesis, teichoic acid and lipoteichoic acid (LTA) synthesis, and cell surface proteins related to epithelial adhesion. Compared with other hosts, strains isolated from sheep and pig contained up to 35 and 33 epithelial adhesion genes, respectively (**Figure 6A**). The significantly distinct genes among strains of each host included cell surface proteins, poly(glycerol-phosphate) alpha-glucosyltransferase, regulation of D-alanyl-lipoteichoic acid biosynthesis (DltR), and the sensor histidine kinase (**Figure 6B**). Other adhesion genes, such as teichoic acid glycosylation protein, LTA synthase, LTA synthesis, DltD, and chaperonin were present uniformly in all 37 strains. Compared with the other isolates, the sheep isolates had the most cell surface protein-encoding genes, with an average of 19, while the gene counts in the goat and horse isolates were the lowest, both of which were an average of 10.

Many surface proteins were predicted to be involved in epithelial adhesion. The gene counts of cell surface proteins were significantly different among the nine hosts. Thus, we collected 17 gene families related to cell surface proteins to indicate which one contributed to the difference (**Figure 6C**). Host-associated lineages were significantly distinguished by genes related to some cell surface proteins, including cell surface protein precursor, cell wall surface anchor family protein, surface protein Rib, and cell-wall-anchored protein SasA (LPXTG motif). Fibronectin binding protein was present in all strains with the same gene count. Compared with other isolates, abundance of the cell surface protein precursor, cell wall surface anchor family protein, and surface protein Rib encoding genes in sheep isolates was highest, with averages of two, one, and four, respectively. The abundance of the cell wall-anchored protein *Sas*A encoding gene was the highest in the sourdough isolates and the putative Staphylococcal surface anchored protein-encoding gene only existed in the sourdough isolates.

### Active Carbohydrate Enzymes

**FIGURE 7:**
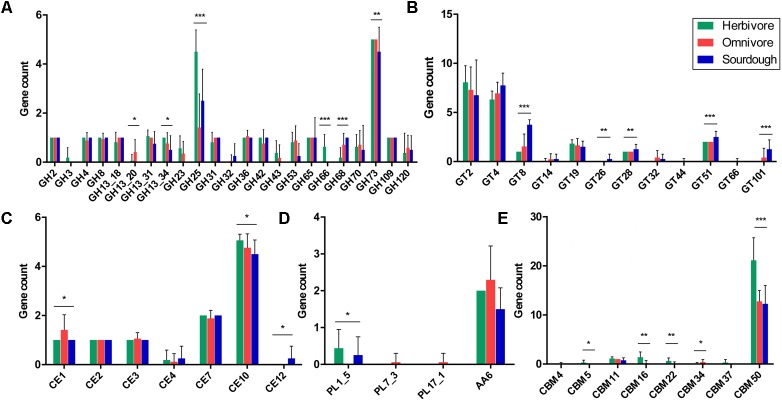
Distribution and abundance of active carbohydrate enzyme family genes in *Lactobacillus reuteri* strains isolated from herbivores, omnivores, and sourdough. **(A)** Glycoside hydrolase (GH) family genes. **(B)** Glycosyltransferase (GT) family genes. **(C)** Carbohydrate esterase (CE) family genes. **(D)** Polysaccharide lyase (PL) and auxiliary activity (AA) family genes. **(E)** Carbohydrate-binding module (CBM) family genes. ^∗^*p* < 0.05, ^∗∗^*p* < 0.01, ^∗∗∗^*p* < 0.001.

Analysis of the 37 genomes revealed that they encoded representatives of 20 of the 148 GH families, with four of the 42 GH13 subfamilies in the CAZy database and a high level of GH-encoding diversity. The abundance of GH13_20, GH13_34, GH25, GH66, GH68, and GH73 differed significantly among strains isolated from the herbivores (sheep, goat, cow, and horse), omnivores (human, pig, mouse, and rat), and sourdough. The GH25 count was the highest in the herbivore isolates, with an average of 4.5, whereas the GH25 count was the lowest in the omnivore isolates, with an average of 1.4. Interestingly, GH3 and GH66 only occurred in the herbivore group, and GH32 was absent from the herbivore group. GH13_20, GH23, and GH43 were absent from the sourdough group (**Figure 7A**).

The 37 genomes collectively encoded 12 of the 105 GT families in the CAZy database. The abundances of GT8, GT26, GT28, GT51, and GT101 differed significantly among the strains isolated from herbivores, omnivores, and sourdough. The GT8 count was lower in the herbivore isolates than that in the omnivore and sourdough isolates (1 vs. 1.5 vs. 3.75), and the GT8 count was the highest in the sourdough isolates. Interestingly, GT32 and GT101 were absent from the herbivore group, and GT26 only existed in the sourdough group (**Figure 7B**).

Furthermore, we identified the CE, PL, AA, and CBM families in 37 isolates from herbivores, omnivores, and sourdough, respectively. A total of 16 CE, 27 PL, 13 AA, and 83 CBM families are in the CAZy database. In the present study, seven CE, three PL, one AA, and eight CBM families were identified in the 37 *L*. *reuteri* isolates. The abundances of CE1, CE10, CE12, PL1_5, CBM5, CBM16, CBM22, CBM34, and CBM50 were significantly different among the three groups. The counts of CBM50 were highest in the herbivore isolates (21 vs. 12) compared with the other two groups. PL1_5 was absent from the omnivore isolates, whereas PL7_3 and PL17_1 were only present in the omnivore isolates. CE12 only existed in the sourdough isolates (**Figures 7C–E**).

## Discussion

This is the first attempt to decipher the genetic features and evolutionary strategies of *L*. *reuteri* in herbivores. We compared the genomic information of *L*. *reuteri* derived from goats, sheep, cows, and horses with that from humans, mice, rats, pigs, and sourdough and revealed the genetic basis for the characteristics that are likely related with the host.

Genome size, G + C content, and the ANI values of the *L*. *reuteri* strains from the different hosts indicated that the strains have broad genetic diversity (**Supplementary Table S2**). Remarkably, the G + C content of the 16 *L*. *reuteri* strains in this study decreased slightly compared to that in the human *L*. *reuteri* strains. Generally, the G + C content of a genome is influenced by selection and mutation involving multiple factors, including a symbiotic lifestyle and the environment (Hao et al., 2012). Therefore, we preliminarily inferred that the genomic differences in the *L*. *reuteri* strains might be associated with their colonized environments. We performed SNP calling to determine the polymorphic sites. In total, 92,270 nucleotide mutation sites were discovered among the 37 *L*. *reuteri* strains. All core genes of herbivore isolates displayed *K*_a_/*K*_s_ ratios <1 (**Figure 5**), suggesting strong purifying selective pressure (negative selection) in the herbivore intestinal environment, which may slow down the process of genomic decay and help to maintain these advantageous features. The core genes of the sourdough *L*. *reuteri* isolates were under positive selection to adapt to the sourdough, and this study also demonstrated that food and intestinal habitats exert different selective pressures related to growth rate and metabolism (Zheng et al., 2015).

A total of 726 core genes were detected in the 37 *L*. *reuteri* genomes isolated from nine hosts, which were mainly encoded essential proteins for metabolism (33.57%) (**Figure 1**). These results suggested these genes are indispensable and constitute the basic framework of the *L*. *reuteri* genome and played important roles in maintaining growth and reproduction. Moreover, a large number of host-specific genes were observed in herbivore group and sourdough group (**Figure 2**). We deduced that genome diversity may be associated with their lifestyle adaptation. Previous reports also showed that bacterial genomes change when they adapt to variable conditions (Walter and Klaenhammer, 2011). An interesting feature of herbivore group was the specific genes involved in porphyrin and chlorophyll metabolism and biosynthesis of amino acids. Chlorophyll is a specific nutrient-rich substance in green plants. Most genes involved in chlorophyll synthesis and metabolism have been reported in photosynthetic bacteria (Yamagata et al., 1998), but none have been reported in lactic acid bacteria (LAB). Omnivore could intake meat to meet their own amino acid needs, but diets of herbivore are mainly leaves, grass, and the other plants. To meet demand of herbivore, intestinal microorganism could use non-protein nitrogen to synthesize amino acids, peptides, and proteins (Allison, 1969). Therefore, *L. reuteri* may be involved in biosynthesis of amino acids in intestines of herbivore.

To gain insight into diversification of the herbivore clades, functional genes were identified that were specific to the three individual clades, including clade A and B, clade C and clade II. Genes involved in atrazine degradation were only detected in the clade A and B. Atrazine (2-chloro-4-ethylamino-6-isopropyl-amino-*s*-triazine) is widely used for weed control during corn and sorghum production (Diana et al., 2010). It is a photosynthesis inhibitor and is far more toxic to plants than animals; however, residual atrazine attached to plants enters and remains in the intestines of herbivores affecting the genetic diversity of the intestinal microflora. In addition, genes involved in beta-lactam resistance were unique to the human clade. We postulate that these unique genes are likely derived from antibiotic resistance bacteria in the human intestine by horizontal transfer. Antibiotics are widely used in humans and animals to fight bacterial infections. However, overuse of antibiotics has led to significant contamination of diverse environments, generating an increasing selective pressure on microorganisms and consequently an increased prevalence of antibiotic resistance among bacteria (Qian et al., 2013). In conclusion, above mentioned results also confirmed that the functions of the genetic features associated with *L*. *reuteri* ecotypes reflect their lifestyle in respective hosts (Frese et al., 2011).

One major prerequisite considered for the selection of probiotic LAB has been their capacity to adhere to animal or human intestinal epithelial cells (Buck et al., 2005). Strains of *L*. *reuteri* have been described to possess strong adhesive ability. Therefore, the type and number of genes associated with epithelial adhesion in *L*. *reuteri* strains from nine different hosts were compared in this study. A total of 261 genes belonging to 11 families related to epithelial adhesion were detected in the 37 *L*. *reuteri* strains. The strains isolated from sheep and pigs contained more epithelial adhesion genes, whereas strains isolated from humans, goats, and horses contained fewer epithelial adhesion genes. Generally, *L*. *reuteri* can be detected in a large subset of animals, but the prevalence of *L*. *reuteri* is much lower in humans, and some evidence shows that this species was more prevalent in human fecal samples in the middle of the last century (Leser et al., 2002; Blaser and Falkow, 2009; Walter and Klaenhammer, 2011). Some studies have suggested that the stratified squamous epithelium in animal intestines plays a key role in the richness of lactobacilli, whereas stratified squamous epithelia are absent from the human gut (Wegmann et al., 2015). We deduced that *L*. *reuteri* strains from the human intestine might have lost some genes related to epithelial adhesion during evolution.

In addition, genes encoding the cell surface protein poly (glycerol-phosphate) alpha-glucosyltransferase and those regulating D-alanyl-lipoteichoic acid biosynthesis (DltR) and sensor histidine kinase were significantly different among strains from each host (**Figure 6B**). In particular, significantly more genes encoded cell surface proteins than other adhesion genes, and those of the sheep strains were significantly more abundant than those in the goat and human strains (**Figure 6C**). Hence, cell surface proteins may contribute to diversification of adhesive ability in different lineages and hosts. Cell surface proteins are involved in epithelial adhesion and biofilm formation. We further analyzed the species and the number of cell surface proteins in the *L*. *reuteri* strains originating from the different hosts. In total, 17 types of genes encoding cell surface proteins were detected in the 37 *L*. *reuteri* strains, and five gene families related to cell surface proteins were significantly different among strains from each host. Among them, the number of genes encoding the surface protein Rib and the cell wall anchored protein SasA (LPXTG motif) were significantly different in the *L*. *reuteri* strains from different hosts. The Rib surface protein was first identified in a type III strain of group B *Streptococcus* that confers protective immunity (Stålhammarcarlemalm et al., 1993). Then, it was confirmed that the Rib-like protein is likely a widely distributed cell surface protein in lactobacilli strains and may play a role in adhesion of these lactobacilli to the host gut (Yang et al., 2017). The cell wall anchor protein *Sas*A is an adhesion-associated protein usually detected in *Staphylococcus aureus*, which may promote colonization (Kukita et al., 2013). These adhesion-encoding genes need to be further verified to determine if they are major factors giving rise to the diversification of the adhesive ability in different host strains.

Carbohydrates are the most widely distributed organic compound in nature, and the main source of energy for all organisms. A large proportion of genes identified were involved in carbohydrate metabolism and carbohydrate transport; therefore, we compared the numbers of genes encoding CAZymes among the strains derived from herbivores, omnivores, and sourdough. The results revealed that representatives of 20 GH, 12 GT, 7 CE, 3 PL, 1 AA, and 8 CBM families in the CAZy database have a high level of diversity (**Figure 7**). Interestingly, GH3 (xylan 1,4-β-xylosidase) and GH66 (dextranase) only existed in the herbivore group, and GH23 (chitinase) was absent from the sourdough group. Xylan 1, 4-β-xylosidase enzymes are mainly responsible for hydrolysis of xylans, which are major components of hemicellulose and widely distributed in plants (Schomburg and Salzmann, 1991). Dextranases (EC 3.2.1.11) are bacterial and fungal enzymes that hydrolyze the predominant 1,6-glycosidic bonds found in dextran, which has been isolated from plant material and possesses a largely linear structure comprised of 95% glucose units linked by glycosidic bonds (Hattori and Ishibashi, 1981; Finnegan et al., 2004). It may be that the long-term plant diet of herbivores generates specificity of the indigenous intestinal *L*. *reuteri* strains. Moreover, chitinases could catalyze the hydrolysis of chitin to *N*-acetylglucosamine. Chitin is an insoluble linear b-1,4-linked polymer of *N*-acetylglucosamine that is a component of the cell walls of fungi and certain algae, and in shells and radulae of mollusks (Leisner et al., 2008). We inferred that the existence of chitinases in animal-originating strains might be related to the presence of a large number of fungi in the animal gut. Chitin barely existed in the sourdough.

In summary, our results indicate that *L. reuteri* strains from different hosts have broad genetic diversity, and the genomic diversity is closely associated with its living environment. In addition, the comparative genomics, a powerful and valuable tool, provided abundant and accurate genomic data in our study. In the future, as more isolates are collected from different animals and environment, further evidence may become available for comparing herbivore isolates of *L. reuteri* to the other original isolates, which would provide a strong indication of the factors that have affected its evolutionary history.

## Accession Number

The sequence information of 16 *L*. *reuteri* strains in this paper has been deposited in the National Center for Biotechnology Information (NCBI), the accession numbers are QGHO00000000, QGHP00000000, QGHQ00000000, QGHR00000000, QGHS00000000, QGHT00000000, QGHU00000000, QGHV00000000, QGHW00000000, QGHX00000000, QGHY00000000, QGHZ00000000, QGIA00000000, QGIB00000000, QGIC00000000, and QGID00000000.

## Author Contributions

ZS and HZ designed the experiments. JY, JcZ, and ZY performed the experiments. JZ and YS analyzed the data. JY and JZ manual drafted the manuscript. All authors read and approved the final manuscript.

## Conflict of Interest Statement

The authors declare that the research was conducted in the absence of any commercial or financial relationships that could be construed as a potential conflict of interest.

## Footnotes

1 http://imedea.uib-csic.es/jspecies/about.html

2 http://bioinfogp.cnb.csic.es/tools/venny/index.html

3 http://www.genome.jp/kaas-bin/kaas_main?mode=partial

4 https://code.google.com/p/kaks-calculator/wiki/KaKs_Calculator

5 http://code.google.com/p/paraat/

6 http://hmmer.org/

## Supplementary Material

FIGURE S1 The average nucleotide identity value of 37 *Lactobacillus reuteri* genomes.

FIGURE S2 Annotation of 37 *Lactobacillus reuteri* genomes by Rapid Annotation using Subsystem Technology tool. **(A)** Functional genes of each genome. **(B)** Distribution of each functional catalog in genomes of 37 strains.

FIGURE S3 Core and pan-genomes of *Lactobacillus reuteri* isolates. The gene accumulation curves show the number of total genes (pan-genome) and conserved genes (core-genome) obtained by adding a new genome to a previous set.

FIGURE S4 The distribution of single nucleotide polymorphisms count per 1000 bp.

TABLE S1 Sample information of 37 *Lactobacillus reuteri* strains.

TABLE S2 General genomic features of 16 *Lactobacillus reuteri* strains.

TABLE S3 Pathway annotation of genes specific to *Lactobacillus reuteri* strains from herbivore, omnivore, and sourdough groups.

TABLE S4 Specific genes of *Lactobacillus reuteri* strains from herbivore, omnivore, and sourdough groups.

TABLE S5 Pathway annotation of genes specific to *Lactobacillus reuteri* strains from clade C and clade A and B.

TABLE S6 Specific genes of *Lactobacillus reuteri* from clade C and clade A and B.

TABLE S7 Pathway annotation of genes specific to *Lactobacillus reuteri* strains from clade A and B and clade II.

TABLE S8 Specific genes of *Lactobacillus reuteri* from clade A and B and clade II.

TABLE S9 Single nucleotide polymorphisms information of 37 *Lactobacillus reuteri* strains.
