# Supplementary figures and images for: Comparative Genomics of the Herbivore Gut Symbiont Lactobacillus reuteri Reveals Genetic Diversity and Lifestyle Adaptation
Source: Front Microbiol. 2018 Jun 4;9:1151. doi: 10.3389/fmicb.2018.01151 (PMC5994480; doi:10.3389/fmicb.2018.01151)

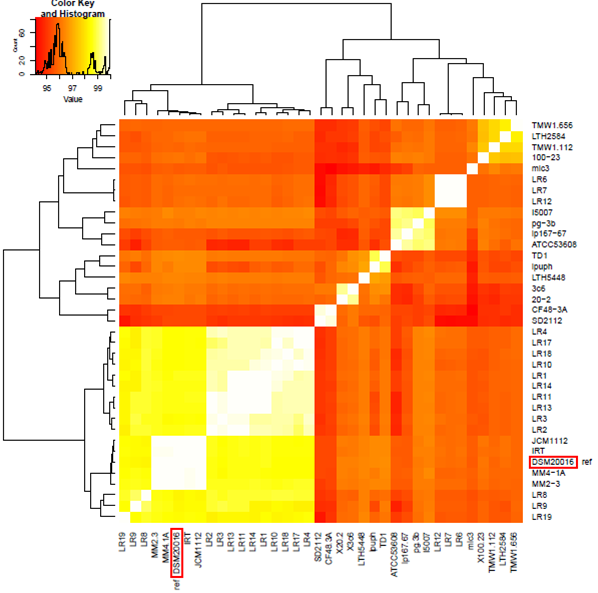

Supplement: FIGURE S1 — The average nucleotide identity value of 37 Lactobacillus reuteri genomes. [file Image_1.TIF]

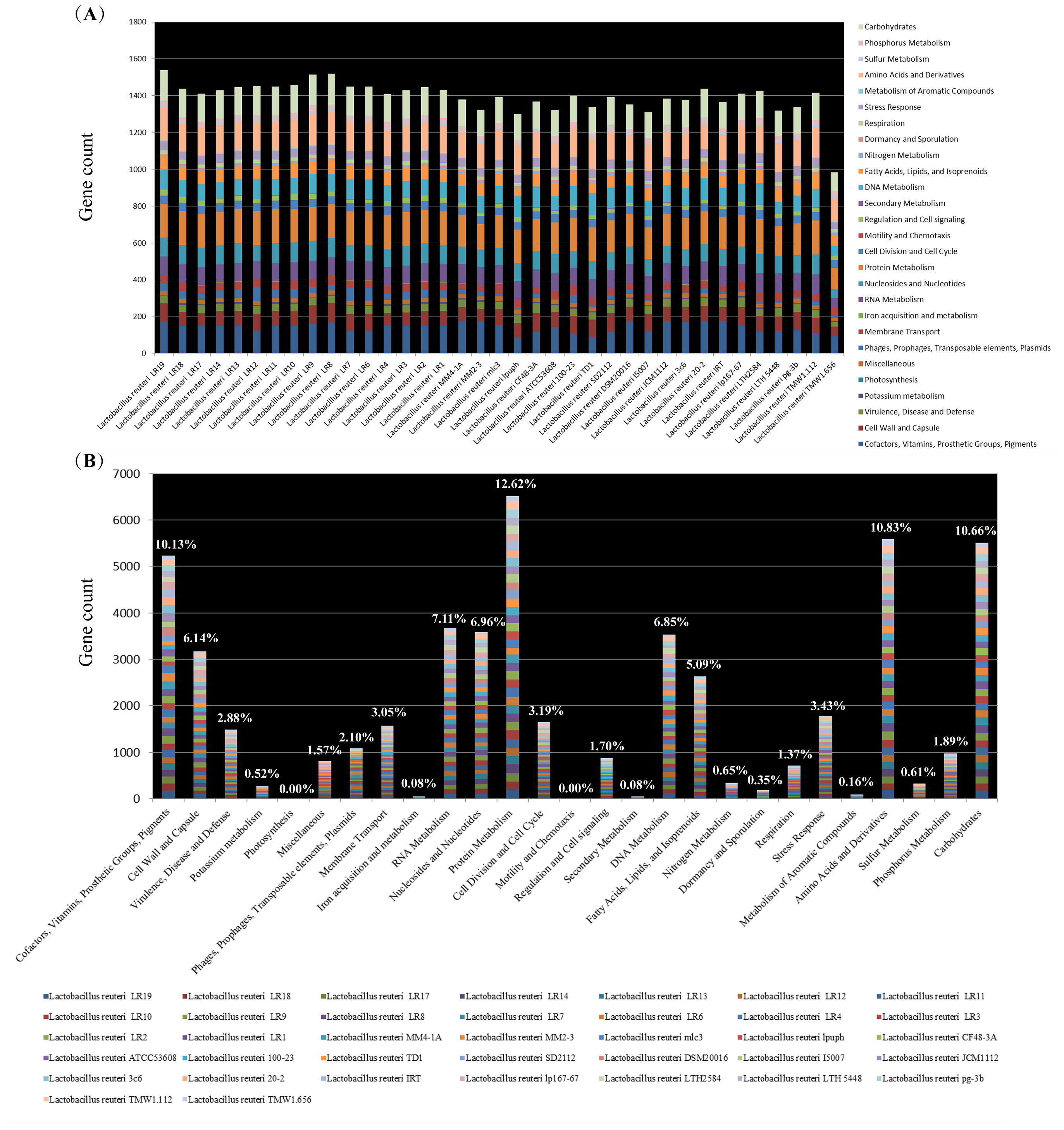

Supplement: FIGURE S2 — Annotation of 37 Lactobacillus reuteri genomes by Rapid Annotation using Subsystem Technology tool. (A) Functional genes of each genome. (B) Distribution of each functional catalog in genomes of 37 strains. [file Image_2.TIF]

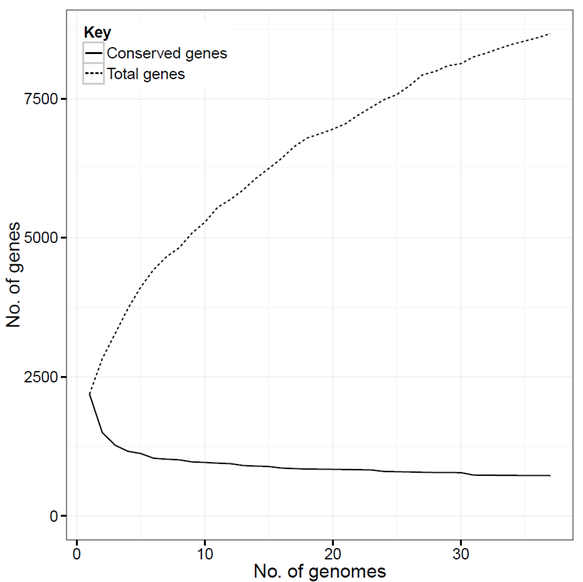

Supplement: FIGURE S3 — Core and pan-genomes of Lactobacillus reuteri isolates. The gene accumulation curves show the number of total genes (pan-genome) and conserved genes (core-genome) obtained by adding a new genome to a previous set. [file Image_3.TIF]

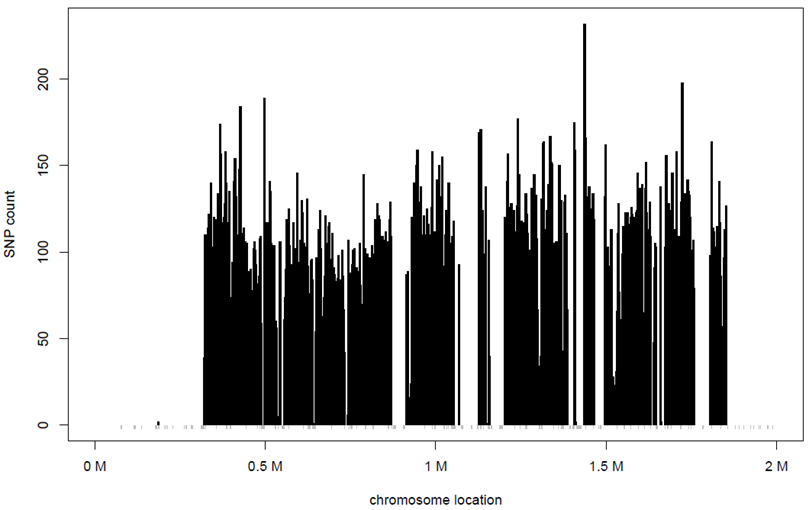

Supplement: FIGURE S4 — The distribution of single nucleotide polymorphisms count per 1000 bp. [file Image_4.TIF]
